# Protocol for monitoring intestinal plasma membrane integrity breached by bacterial pore-forming toxin in *Caenorhabditis elegans*

**DOI:** 10.1016/j.xpro.2026.104679

**Published:** 2026-07-01

**Authors:** Hui-Chen Hsieh, Tzu-Hsuan Chiu, Chang-Shi Chen

**Affiliations:** 1 Department of Biochemistry and Molecular Biology, College of Medicine, National Cheng Kung University, Tainan, Taiwan; 2 Institute of Basic Medical Sciences, College of Medicine, National Cheng Kung University, Tainan, Taiwan

**Keywords:** Cell Membrane, Microbiology, Model Organisms

## Abstract

Plasma membrane integrity (PMI) safeguards cellular homeostasis but can be disrupted by bacterial pore-forming toxins (PFTs), necessitating complete pore repair for host survival. Here, we present an *in vivo* protocol for monitoring apical plasma membrane perforation and its restoration in intestinal epithelial cells of *C. elegans* following intoxication with the PFT Cry5B. We describe steps for utilizing membrane-impermeable dyes, such as propidium iodide (PI), as a functional readout to assess PMI and quantitatively evaluate pore-repair capacity in *C. elegans*.

For complete details on the use and execution of this protocol, please refer to Hsieh, HC et al.^1^

## Highlights

• Protocol to detect intestinal epithelial PMI disrupted by bacterial PFTs in *C. elegans*
• Workflow of PFT-induced plasma membrane perforation in *C. elegans*
• *In vivo* staining using membrane-impermeable dye in *C. elegans*
• Real-time *in vivo* imaging of pore formation and repair at organismal resolution

## Abstract (editor highlights)

Publisher’s note: Undertaking any experimental protocol requires adherence to local institutional guidelines for laboratory safety and ethics.

## Graphical abstract

**Figure:**
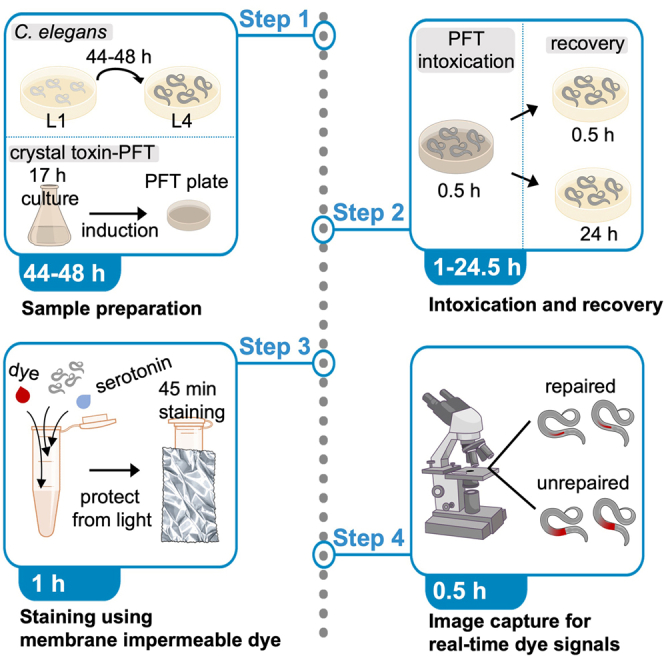

## Before you begin

**Figure 1:**
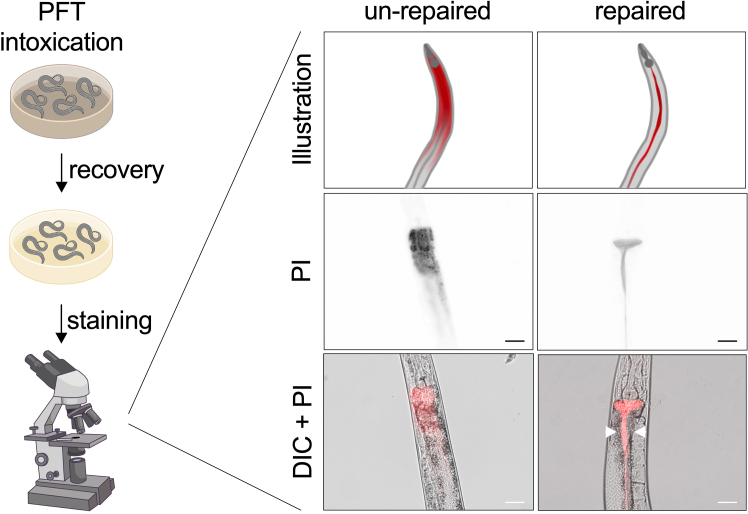
The diagram of detection for plasma membrane integrity in response to PFT intoxication We expose *C. elegans* to PFTs for 30 min and then transfer them to a non-pathogenic *E. coli* plate to recover for the indicated time. After recovery, we stain the animals with membrane-impermeable propidium iodide (PI) for 45 min and capture the images of real-time dye signal using a fluorescence microscope. Scale bar: 20 μm. DIC indicates differential interference contrast. Illustration shows conceptual results for repaired and un-repaired signals.

Plasma membrane integrity (PMI) functions as the primary barrier against environmental insults, such as pathogen infection and mechanical stress. Once PMI is disrupted, cells lose cellular homeostasis, which is detrimental to survival. The bacterial pore-forming toxin (PFT) family is among the most common virulence factors produced by pathogens. PFTs, such as crystal toxins generated by *Bacillus thuringiensis* (*Bt*), streptolysin O (SLO) from *Streptococcus pyogenes*, and hemolysin produced by *Aeromonas dhakensis*, are well known for their ability to perforate host cell plasma membranes, disrupting PMI and ultimately leading to fatal outcomes for the host. Therefore, the host must restore the PMI in response to intoxication of PFTs. To investigate plasma membrane pores in intestinal cells at organismal resolution, we developed an *in vivo* protocol to monitor apical plasma membrane perforation and restoration in intestinal epithelial cells of *C. elegans* (Figure 1). We use representative PFTs-crystal toxin Cry5B from *Bt* to perforate the cell plasma membrane, and membrane-impermeable propidium iodide as an indicator of PMI. This *in vivo* pore repair assay was initially developed by Los, F. C. *et al.*^2^ and subsequently revised and applied in our previous publications to evaluate the plasma membrane repair ability of *C. elegans* in response to various bacterial PFTs.^1^^,^^3^

### Innovation

This protocol is user-friendly and provides a straightforward, intuitive approach to evaluate plasma membrane repair in response to PFTs in *C. elegans*. Users can observe the effects of pore formation and repair after PFT exposure by feeding transparent *C. elegans* a membrane-impermeable dye, then detecting fluorescence. The innovation of this protocol is that, using *C. elegans* as an animal model, it provides an organismal-level evaluation of plasma membrane pore repair in response to bacterial PFTs intoxication.

### Culturing *C. elegans* stage 4 larvae for the experiment

Maintaining *C. elegans* on an ENG plate with an OP50 lawn at an adequate temperature until the gravid adult stage. For wild-type N2, L1 larvae were cultured at 20°C for 3 days until they began carrying eggs and acquire eggs from gravid adult worms by bleaching. We have listed the materials we used in bleaching in the materials and equipment section. For the detailed bleaching protocol, please refer to Step 2 in step-by-step method details section. After 12–16 h, seeding the hatched L1 larvae on the ENG plate with an OP50 bacterial lawn. It takes 44–48 h at 20°C for wild-type N2 worms to reach the L4 larval stage. At this stage, the worms are ready for bacterial PFT intoxication, followed by the plasma membrane repair assay.

### ENG-IC plate preparation

For crystal toxin, Cry5B-PFT, generation, we use Enriched Nematode Growth medium with isopropyl β-D-1-thiogalactopyranoside (IPTG) and carbenicillin, abbreviated as ENG-IC, to induce pQE9-Cry5B protein expression. One liter of ENG consists of 0.5% bactopeptone, 0.1% yeast extract, 51 mM NaCl, and 3% agar. After autoclaving, add to a final concentration of 5 μg/ml cholesterol in ethanol, 1 mM CaCl_2_, 1 mM MgSO_4_, and 25 mM KPO_4_ buffer (119.35 g KH_2_PO_4_, 21.43 g K_2_HPO_4_, H_2_O to 1 liter, pH=6.0). For ENG-IC, we supplemented the ENG solution with 50 μM IPTG and 50 μg/mL carbenicillin. After thoroughly mixing, we dispense the solution into petri plates, filling each plate two-thirds full with agar, and store the ENG-IC plates at 4°C. We also list the formula of ENG and ENG-IC plates in materials and equipment section.

***Note:*** ENG-IC plates stored for more than 3 months may expire.

### 2% agarose pads

For signal detection of membrane-impermeable dye, worms are placed on a glass slide with 2% agarose and covered with a coverslip for observation. We prepare agarose pads by dissolving 2% agarose in M9 buffer, heating the mixture in a microwave until fully melted, then placing a drop of molten agarose on a glass slide and immediately overlaying it with a second slide to flatten the agarose. After cooling and solidification, we gently removed the top slide for use.

## Key resources table

**Table:** 

REAGENT or RESOURCE	SOURCE	IDENTIFIER
**Bacterial strains**		

*E. coli*. Uracil auxotroph, standard *C. elegans* laboratory food source.	*Caenorhabditis* Genetics Center (CGC)	Strain: OP50
Streptomycin resistant strain of *E. coli* OP50	*Caenorhabditis* Genetics Center (CGC)	Strain: OP50-1
*E. coli* OP50 carrying pQE9-Cry5B	This study^1^^,^^3^	Strain: YQ603

**Chemicals, peptides, and recombinant proteins**		

PEPTONE (Bacteriological)	BioShop	Cat#: PEP403.500
Yeast Extract	thermo scientific	Cat#: J23547-A1
Sodium chloride (NaCl)	Honeywell	Cat#: 31434
Difco™ Agar	BD	Cat#: 214010
Magnesium sulfate heptahydrate (MgSO_4_)	Sigma-Aldrich	Cat#: M2773
Calcium chloride dihydrate (CaCl_2_)	Merk	Cat#: 10035-04-8
Cholesterol	Sigma-Aldrich	Cat#: C8503
Potassium Phosphate (KH_2_PO_4_)	J.T.Baker	Cat#: 3246-05
Potassium Phosphate (K_2_HPO_4_)	J.T.Baker	Cat#: 3252-05
Sodium hydrogen phosphate heptahydrate (Na_2_HPO_4_)	thermo scientific	Cat#: 011592.36
Sodium hypochlorite solution, 6%–14% active chlorine basis (bleach)	Honeywell	Cat#: 13440
Potassium hydroxide (KOH)	Sigma-Aldrich	Cat#: P5958
Carbenicillin	Sigma-Aldrich	Cat#: 4800946
Isopropyl-β-D-1-thiogalactopyranoside (IPTG)	Cyrusbioscience or Sigma-Aldrich	Cat#: 101-367-93-1 for Cyrusbioscience Cat#: I6758 for Sigma-Aldrich
Propidium iodide (PI)^a^	Sigma-Aldrich	Cat#: 25535164
Serotonin	Sigma-Aldrich	Cat#: 61-47-2
Streptomycin sulfate salt	Sigma-Aldrich	Cat#: S9137
Sodium azide	KANTO CHEMICAL CO., INC.	Cat#: 37824-25

**Deposited data**		

All image data	This study	BioStudies: accession number S-BSST2890.

**Experimental models: Organisms/strains**		

*C. elegans:* wild type, hermaphrodite, late L4 stage	Caenorhabditis Genetics Center (CGC)^4^	Strain: Bristol N2^5^

a PI is a DNA-intercalating fluorescent dye which should be handled as a potentially mutagenic and hazardous reagent. Appropriate personal protective equipment, waste disposal and institutional safety procedures should be followed.

## Materials and equipment

**Table:** Enriched Nematode Growth medium (ENG) and ENG-IC

Reagent	Final concentration	Amount
peptone	0.5%	5 g
yeast extract	0.1%	1 g
NaCl	51 mM	3 g
Difco™ Agar	3%	30 g
ddH2O	N/A	972 mL
MgSO_4_ (1M)	1 mM	1 mL
CaCl_2_ (1 M)	1 mM	1 mL
phosphate buffer (1M)	1 mM	25 mL
cholesterol (5 mg/mL in 100% EtOH)	5 μg/mL	1 mL
**Total**	**N/A**	**1000 mL**

Autoclaved. Sterile MgSO_4_, CaCl_2_, phosphate buffer and cholesterol are added after autoclaving.

For ENG-IC, add sterile 1 mL of 50 mg/mL carbenicillin and 1 mL of 50 mM IPTG into 1000 mL ENG.

**Table:** Phosphate Buffer (1M)

Reagent	Final concentration	Amount
KH_2_PO_4_	0.877 M	119.38 g
K_2_HPO_4_	0.123 M	21.43 g
ddH_2_O	N/A	1000 mL
**Total**	**N/A**	**1000 mL**

Adjust the pH to 6.0, followed by autoclaving.

**Table:** 50 mg/mL carbenicillin stock

Reagent	Final concentration	Amount
carbenicillin	50 mg/mL	500 mg
ddH_2_O	N/A	10 mL
**Total**	**N/A**	**10 mL**

Sterilize by passing the solution through a 0.22 μm filter.

**Table:** 50 mM IPTG stock

Reagent	Final concentration	Amount
IPTG	50 mM	119 mg
ddH_2_O	N/A	10 mL
**Total**	**N/A**	**10 mL**

Sterilize by passing the solution through a 0.22 μm filter.

**Table:** 5M KOH

Reagent	Final concentration	Amount
KOH	5M	14.03 g
ddH_2_O	N/A	50 mL
**Total**	**N/A**	**50 mL**

**Table:** M9 buffer

Reagent	Final concentration	Amount
KH_2_PO_4_	22 mM	1.5 g
Na_2_HPO_4_	42 mM	5.66 g
NaCl	85.5 mM	2.5 g
ddH_2_O	N/A	499.5 mL
MgSO_4_ (1M)	1 mM	0.5 mL
**Total**	**N/A**	**500 mL**

Autoclaved. MgSO_4_ is added after autoclaving.

Store at room temperature.

**Table:** Propidium iodide mixture

Reagent	Final concentration	Amount
Propidium iodide (6.7 mg/mL)^a^	6.7 μg/mL	1 μL
Serotonin (10 mg/mL)^b^	5 mg/mL	500 μL
M9	N/A	499 μL
**Total**	**N/A**	**1 mL**

a Propidium iodide (6.7 mg/mL) solution is dissolved in M9 and stored at −4°C in the dark for up to 3 months.

b Serotonin (10 mg/mL) solution should be freshly prepared in M9 buffer just before use. Both the solution and stock should be stored at 4°C in the dark.

## Step-by-step method details

### Worm preparation: Culture synchronized *C. elegans* from the L1–L4 stages

**Timing: 1 week**

In this step, we cultivate *C. elegans* from eggs to the synchronized L4 stage, which is ready for the pore-repair test.

***Note:*** The late L4 stage, which takes around 48 h from the L1 stage in wild-type N2 worms, is better for the assay.

1. Chunk dauer worms onto 6 cm ENG plates with an OP50 lawn and grow to the gravid adult stage.
2. Obtain eggs from gravid adults by bleaching:
  a. Wash worms from ENG plates with sterile ddH_2_O by glass Pasteur pipette and collect them into 15 mL conical centrifuge tube.
  b. Add sterile ddH_2_O to total 15 mL and centrifuge at 500 × *g* for 1 min.
  c. Remove the supernatant by suction.
  d. Repeat the previous two steps (step 2b and step 2c) until the supernatant is clear.
  e. Add sterile ddH_2_O to total 3.5 mL.
  f. Sequentially add 1 mL sodium hypochlorite solution (bleach) first and then 0.5 mL 5M KOH into the conical centrifuge tube.
  g. Secure the cap and keep shaking the tube by putting on the rocker for 4 min.
  h. After 4 min, observe worm body using microscope and repeat shaking until worm body breaks into a quarter. (but do NOT exceed 6 min after adding the bleach and KOH solution)
  i. Stop bleaching by adding sterile ddH_2_O to total 15 mL and centrifuge at 1200 × *g* for 2 min.
  j. Remove the supernatant by suction.
  k. Add sterile ddH_2_O to total 15 mL and centrifuge at 1200 × *g* for 2 min.
  l. Remove the supernatant by suction.
  m. Repeat the previous two steps (step 2k and step 2l) twice.
  n. Remove the supernatant as much as possible by suction.
  o. Suspend the eggs with 500 μL M9 in BSC and transfer the egg solution into 3.5 cm petri dish containing 500 μL M9.
  p. Thoroughly mix the egg solution with gentle pipetting and make sure the solution spread evenly in the 3.5 cm petri dish.
3. Resuspend the eggs in M9 buffer and incubate the eggs at 20°C for 12–16 h until hatching to synchronize L1 larvae.
4. Seed around 2000 synchronized population of L1 larvae onto a 6 cm ENG plate and incubate at 20°C.
5. Grow the worms to the synchronized L4 stage.

***Note:*** The bleaching protocol is adopted and revised from Stiernagle T.^6^ We recommend stopping the bleaching step by adding ddH_2_O as soon as the worm body is broken down to approximately one-quarter of its original size to avoid a low hatching rate.

***Note:*** Collect the worms using a sterile glass Pasteur pipette. Avoid transferring worms with a plastic tip or dropper, as they tend to adhere to the plastic interior. If a plastic tip must be used, pre-rinsing it with 1% Triton X solution can help prevent sticking.

### Crystal toxin plate preparation: Induce Cry5B-PFT protein expression

**Timing: 2 days**

This section describes the preparation of Cry5B-PFT plates through the cultivation and induction of *E. coli* OP50 harboring the pQE9-Cry5B plasmid.

6. Culture pQE9-Cry5B expressing bacteria broth:
  a. Pick one colony from the streaking plate using a sterile toothpick, then transfer it into 3 mL LB broth containing 50 μg/mL carbenicillin.
  b. Culture the bacterial broth in a 37°C incubator with shaking at 210 rpm for 16–18 h.

***Note:*** Streak a new bacterial plate from stock every month to ensure toxin activity.

7. Refresh bacterial broth:
  a. Dilute 1 mL of overnight-cultured bacterial broth with 4 mL LB medium containing 50 μg/mL carbenicillin.
  b. Incubate the bacterial broth in a 37°C incubator with shaking at 210 rpm for 1 h to refresh.

***Note:*** The OD_600_ of refreshed bacterial broth should be 0.6–0.8.

8. pQE9-Cry5B protein induction:
  a. Add IPTG to the refreshed bacterial broth at a final concentration of 50 μM and culture at 30°C with shaking at 210 rpm for 3 h.
9. Spread bacterial lawn:
  a. Adjust the OD_600_ to 1.95–2.05.
  b. Apply 30 μL bacterial broth (OD_600_ = 1.95–2.05) to the ENG-IC plate.
  c. Incubate the plate at 25°C overnight.

***Note:*** The plate should be used within 3 days.

### Pulse *C. elegans* on Cry5B-PFT: Perforate intestinal epithelial cells by Cry5B intoxication

**Timing: 0.5–1 h**

In this section, we expose L4-stage *C. elegans* to Cry5B-PFT plates to induce intestinal epithelial cell perforation.

10. Transfer L4 larvae using a platinum picker from ENG to an ENG-IC plate with crystal toxin, or wash worms from ENG and seed onto crystal toxin plates if needed; at least 100 worms per plate.
11. Feed worms with crystal toxin Cry5B for 30 min at 25°C for intoxication.
12. Resuspend intoxicated worms from crystal toxin plates with 1 mL sterile M9 and collect them into a 1.5 mL microcentrifuge tube.
13. Centrifuge at 500 × *g* for 1 min.
14. Remove supernatant by suction.
15. Wash the worms with 1.5 mL sterile M9 and centrifuge at 500 × *g* for 1 min.
16. Remove the supernatant by suction.
17. Repeat the previous two steps (step 15 and step 16) two times.

***Note:*** To minimize toxin carryover, for step 12, the plates are washed with M9 at most two times to collect the animals.

***Note:*** To remove bacteria as complete as possible, for step 17, if the supernatant remains cloudy after two washes, continue repeating step 15 and step 16 until the supernatant is clear.

### Transfer *C. elegans* to non-pathogenic *E. coli*: Recover from plasma membrane perforation

**Timing: 0.5–24 h**

In this section, we transfer intoxicated *C. elegans* to non-pathogenic *E. coli* OP50 bacteria lawn for recovery from the plasma membrane disruption.

18. Seed the washed worms onto two 6 cm ENG plates with 50 μg/mL streptomycin and non-pathogenic *E. coli* OP50 bacteria lawn; at least 40 worms per plate. One plate for 30 min recovery, another for 24 h recovery.
19. Incubate the worms at 20°C for 30 min and 24 h recovery respectively.

***Note:*** ENG plates containing 50 μg/mL streptomycin are used to eliminate OP50-pQE9-Cry5B to achieve a maximal pore repair rate.

***Note:*** 30 min recovery for confirming pore formation and 24 h for confirming pore repair.

### Membrane-impermeable dye staining: Assess plasma membrane integrity

**Timing: 1 h**

We stain recovered *C. elegans* using membrane impermeable dye that enters the cytosol of intestinal cells in unrepaired animals.

20. Resuspend the recovered worms from the ENG plate in sterile 1 mL M9 and collect them into a 1.5 mL microcentrifuge tube.
21. Centrifuge at 500 × *g* for 1 min, then remove the supernatant.
22. Add sterile M9 containing 5 mg/mL serotonin and 6.7 μg/mL PI to a total of 500 μL in the 1.5 mL microcentrifuge tube.
23. Incubate at 25°C with gentle shaking for 45 min; keep protected from light.
24. Centrifuge at 500 × *g* for 1 min, then remove the supernatant.
25. Add sterile M9 to a final volume of 1.5 mL, then centrifuge at 500 × *g* for 1 min.
26. Remove the supernatant by suction.
27. Repeat the previous two steps twice for the wash.

***Note:*** The serotonin solution should be freshly prepared just before use and protected from light.

***Note:*** Membrane impermeable dye should be protected from light.

### Image capture: Evaluate plasma membrane integrity through membrane-impermeable dye signal distribution

**Timing: 0.5–1 h**

In this step, we capture the real-time *in vivo* membrane impermeable dye signal in *C. elegans* through microscope system.

28. Seed the washed worms onto a 2% agarose slide and let the remaining M9 slightly absorb into the agarose.
29. Add 3 μl 3% sodium azide to the worms on 2% agarose.
30. Arrange worms as they gradually become paralyzed.
31. Apply the coverslip.
32. Capture fluorescence signal (RFP for PI) and differential interference contrast image (DIC) using a 40× objective lens by Nikon Eclipse Ti inverted microscope system (Figure 1).

***Note:*** For statistics, calculate at least 30 worms for each group.

***Note:*** The coverslip naturally adheres to the agarose due to surface tension induced by the sodium azide solution.

### Statistical analysis

**Timing: 0.5 h**

After image acquisition, we calculate the percentage of animals with repaired plasma membranes and perform statistical analyses between groups.

33. Calculate the percentage of animals with plasma membrane pore repair separately after 30 min and 24 h recovery; at least 30 animals per group.
34. Analyze the data using Prism GraphPad.
35. Select ‘‘column’’, appearance: violin plot. Show all data points.
36. For two groups (one control and one experimental group), use unpaired *t* test (two-tailed) for statistical analysis; for more than two groups, use two-way ANOVA.

## Expected outcomes

**Figure 2:**
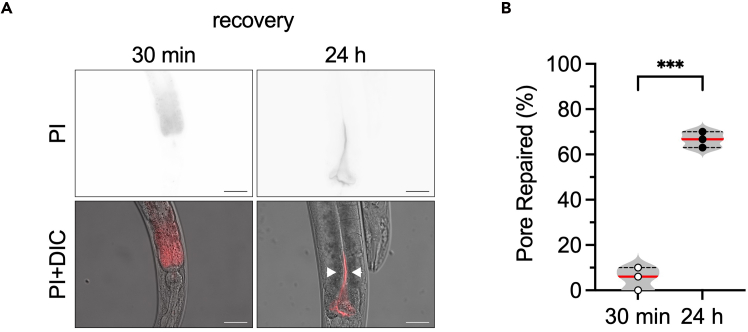
The expected outcomes of *C. elegans* exposed to PFT, followed by recovery on non-pathogenic *E. coli* for either 30 min or 24 h (A) The representative images of worms intoxicated by PFT for 30 min and then transferred to non-pathogenic *E. coli* for either 30 min or 24 h recovery. PI indicates propidium iodide. DIC represents differential interference contrast. Scale bar: 20 μm. (B) The quantification of worms exposed to PFT for 30 min followed by 30 min or 24 h recovery. Red bar indicates mean value. ∗∗∗*p* < 0.001.

In this protocol, we systematically perform *C. elegans* preparation, Cry5B-PFT induction, pore formation, pore repair, and subsequent image analysis. Upon Cry5B exposure, membrane-impermeable dye staining is anticipated to reveal compromised intestinal plasma membrane integrity, as indicated by increased dye penetration into intestinal cells. This outcome reflects effective pore formation triggered by PFT intoxication. At the same time, *C. elegans* with pore-repair ability are expected to exhibit confined dye signals in the intestinal lumen after 24 h of recovery, suggesting successful restoration of plasma membrane integrity (Figures 2A and 2B). By comparing the control and Cry5B groups and across different genetic backgrounds, this protocol enables an organismal-level assessment of intestinal plasma membrane integrity and the evaluation of PFT-induced pore formation and repair dynamics.

## Limitations

Several limitations should be considered. First, membrane-impermeable dye uptake serves as an indirect indicator of plasma membrane integrity and may not reflect solely pore formation by Cry5B-PFT. Other forms of membrane perturbation or physiological stress may also contribute to increased permeability. Second, there is variability in membrane-impermeable dye uptake among individual worms, especially in worms with pore-repaired defects. Also, differences in ingestion, developmental stage, and physiological state may contribute to heterogeneity in dye uptake.

Taken together, while this protocol enables organismal-level assessment of intestinal plasma membrane integrity and PFT-induced pore formation, results should be interpreted with these limitations in mind.

## Troubleshooting

### Problem 1

*Unsuccessful staining* (related to Step 20–27).

### Potential solution

**Figure 3:**
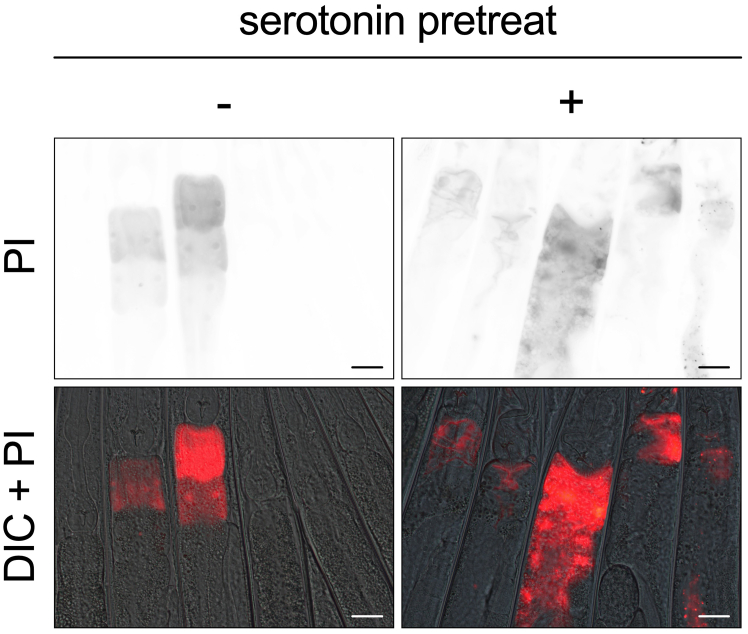
The representative images of poor staining and improved results Serotonin pretreatment prior to dye staining enhances pharyngeal pumping in *C. elegans*, resulting in improved staining compared to untreated animals. Scale bar: 20 μm. PI denotes propidium iodide. DIC indicates differential interference contrast.

Poor staining may be caused by inefficient pharyngeal pumping, which often occurs in unrepaired worms after 24 h of recovery. Therefore, we promote pharyngeal pumping by pretreating the worms with serotonin, which stimulates muscle contraction. Unlike the described protocol, the recovered worms are soaked in 5 mg/mL serotonin for 30 min to increase the pumping rate. Membrane-impermeable dye is then added to the solution for staining. The outcome is indicated in Figure 3. In addition to serotonin pretreatment, we also suggest adjusting serotonin and dye concentration as well as staining time.

### Problem 2

*Unsuccessful plasma membrane repair* (related to Step 12–17).

### Potential solution

Failed plasma membrane repair may result from PFT contamination. During worm collection from crystal toxin plates, the plates are washed with M9 no more than two times to minimize toxin carryover. To ensure sufficient clean samples, more than 300 worms are seeded per plate for intoxication. After collection via two M9 washes from crystal toxin plates, the remaining worms on the plates are discarded. We recommend multiple washes until the supernatant is clear in the microcentrifuge tube.

## Resource availability

### Lead contact

Further information and requests for resources and reagents should be directed to and will be fulfilled by the lead contact, Chang-Shi Chen (cschen@mail.ncku.edu.tw).

### Technical contact

Technical questions on executing this protocol should be directed to and will be answered by the technical contacts, Tzu-Hsuan Chiu (s16144012@gs.ncku.edu.tw) and Hui-Chen Hsieh (s16074055@gs.ncku.edu.tw).

### Materials availability

This study did not generate new reagents.

### Data and code availability

Original data have been deposited to BioStudies (www.ebi.ac.uk/biostudies/studies) under accession no. S-BSST2890.

## Acknowledgments

This work was supported by the 10.13039/100020595National Science and Technology Council (10.13039/100020595NSTC) grants, NSTC 113-2811-B-006-051 to H.-C.H. and NSTC 113-2320-B-006-024-MY3 to C.-S.C. The funder had no role in study design, data collection and analysis, the decision to publish, or the preparation of the manuscript.

## Author contributions

H.-C.H. and C.-S.C. conceived and designed the protocols. H.-C.H., T.-H.C., and C.-S.C. wrote the paper.

## Declaration of interests

The authors declare no competing interests.
